# Cryopreservation of 3D Bioprinted Scaffolds with Temperature-Controlled-Cryoprinting

**DOI:** 10.3390/gels9060502

**Published:** 2023-06-20

**Authors:** Linnea Warburton, Boris Rubinsky

**Affiliations:** 1 Department of Mechanical Engineering, University of California Berkeley, Berkeley, CA 94720, USA; 2 Department of Bioengineering, University of California Berkeley, Berkeley, CA 94720, USA

**Keywords:** 3D bioprinting, cryopreservation, 3D cell culture, alginate, freezing

## Abstract

Temperature-Controlled-Cryoprinting (TCC) is a new 3D bioprinting technology that allows for the fabrication and cryopreservation of complex and large cell-laden scaffolds. During TCC, bioink is deposited on a freezing plate that descends further into a cooling bath, keeping the temperature at the nozzle constant. To demonstrate the effectiveness of TCC, we used it to fabricate and cryopreserve cell-laden 3D alginate-based scaffolds with high cell viability and no size limitations. Our results show that Vero cells in a 3D TCC bioprinted scaffold can survive cryopreservation with a viability of 71%, and cell viability does not decrease as higher layers are printed. In contrast, previous methods had either low cell viability or decreasing efficacy for tall or thick scaffolds. We used an optimal temperature profile for freezing during 3D printing using the two-step interrupted cryopreservation method and evaluated drops in cell viability during the various stages of TCC. Our findings suggest that TCC has significant potential for advancing 3D cell culture and tissue engineering.

## 1. Introduction

**Figure 1:**
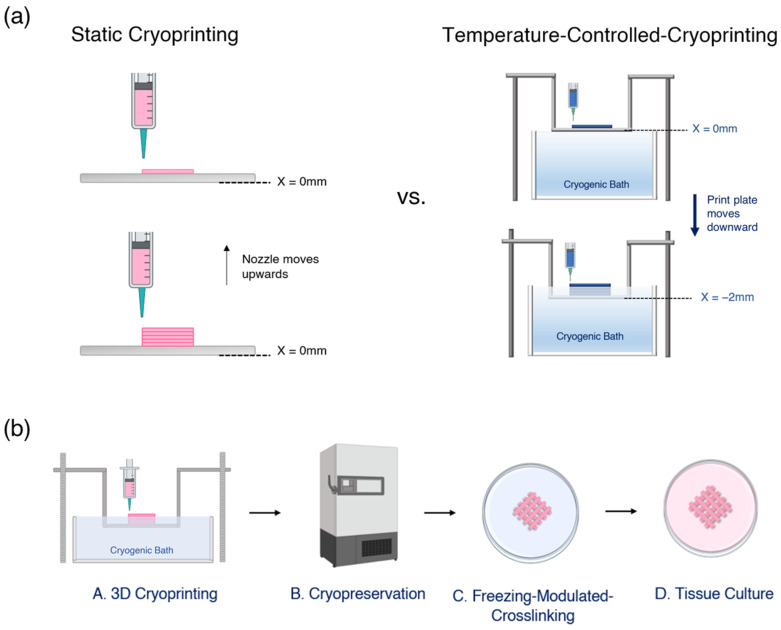
(**a**) Static cryoprinting versus Temperature-Controlled Cryoprinting. (**b**) A supply chain of cell-laden scaffolds from fabrication to use.

Three-dimensional bioprinting has the potential to revolutionize tissue engineering in numerous applications, including drug testing, tissue repair, and organ replacement. As tissue engineering advances and products are manufactured on an industrial scale, there is a growing need to cryopreserve 3D bioprinted scaffolds [1]. Historically, cryopreservation has been the main approach for creating stores of cells, tissue constructs, and even whole organs. Cell-laden 3D scaffolds could be cryopreserved, shipped to laboratories, and then thawed when needed (See Figure 1b). Such a process would dramatically reduce the time and labor associated with 3D bioprinted scaffolds and expand their use to laboratories that do not have the resources to fabricate them in-house.

Decades of research have established the optimal cryopreservation protocols for individual cells [2,3,4,5] However, the ubiquitous methods used to cryopreserve cells in a medium are often ineffective for 3D bioprinted scaffolds. There exists a significant gap in the literature when it comes to cryopreserving 3D bioprinted scaffolds, and current approaches have had limited success [6]. The logical approach is to 3D bioprint a cell-laden scaffold and then later freeze it, however, there are two major issues with this approach [1]. The first issue is that freezing from outside inward creates an uneven temperature gradient throughout the scaffold such that the cells are frozen at different rates. It is well established that cell survival during cryopreservation is dependent on the temperature history the cells experience during freezing. The second issue is the non-uniform distribution of cryoprotectants [7]. Cryoprotectants are chemicals that are added to the cell medium to reduce cell death during freezing. When cryoprotectants are introduced from the exterior of the object, cells deep in the scaffold risk being exposed to insufficient levels of cryoprotectant, and cells at the surface of the scaffold risk death from cryoprotectant toxicity. Both of these issues become magnified the larger the size of the scaffold. Although there have been some successes in cryopreserving tissue-engineered scaffolds, cell viability was often below 50% and the size of the scaffolds was often less than 0.15 cm^3^ [1].

Rather than freezing a finished scaffold, a more promising approach is to combine the two steps of 3D printing and cryopreservation into one step [8]. For example, Ravanbakhsh used cryoprinting to cryopreserve cell-laden scaffolds [9], Luo et al. used cryoprinting to print freestanding filamentous constructs that mimicked the muscle–tendon unit [10]. These approaches can be classified as “static” cryoprinting, as they involve printing onto a freezing plate [11], (See Figure 1a). A notable limitation of static cryoprinting is that as each layer is printed, the last printed layer moves further away from the freezing plate and so the temperature of the last printed layer rises. As noted by Ravanbakhsh et al., 2022 [12], the reduced heat transfer rate as printing continues prevents the printing of thick constructs and leads to cell death in the higher layers. This limitation of static 3D cryoprinting is eliminated by temperature-controlled cryoprinting [8] (Figure 1b).

In this paper, we present “Temperature Controlled Cryoprinting” (TCC) as a method of fabricating and cryopreserving 3D bioprinted scaffolds. Temperature-controlled cryoprinting uses a bath of cooling fluid to cool the print plate and, as each layer is printed, the print plate descends further into the cooling bath by the height of the layer (See Figure 1a). As a result, the lower layers of the printed scaffold become immersed in the cooling fluid and the cooling rate during freezing of each new layer is kept constant. We demonstrate that Vero cells in an alginate–collagen bioink can survive temperature-controlled cryoprinting and cryopreservation at −80 °C with a viability of 71.64 ± 7.47% in multi-layer scaffolds. Additionally, we find that cell viability does not decrease as higher layers are printed, demonstrating the merits of TCC versus static cryoprinting. Vero cells were used as a test bed because of their widespread use as a host for studying viruses [13]. An effective cryopreservation process for 3D scaffolds laden with Vero cells would allow virology researchers to create a stockpile of cryopreserved scaffolds that could be thawed at any time and infected with a virus.

There are two other advantages of TCC that we have demonstrated in prior work with acellular hydrogels. The first is the ability to print large structures out of soft bioink without them collapsing [8,14]. Within 3D bioprinting, it remains difficult to print complex structures or overhangs out of the soft bioink that are meant to mimic soft tissue. Previously, static cryoprinting has been used to freeze and thus stabilize bioink as they are deposited [10,15]. However, the reduced heat transfer rate as printing advances from the printing surface prevents the printing of thick constructs [12]. In contrast, TCC allows for the freezing of each 3D-printed voxel layer with controlled cooling rates, independent of the height of the structure [8,14,16,17]. Previously, we used TCC to print multi-layer structures out of acellular alginate and developed a crosslinking technique called freezing-modulated crosslinking to crosslink the objects as they thawed [16]. In this study, we build upon those results by printing eight freestanding layers of cell-laden alginate without a support bath or the use of sacrificial materials.

The second and final advantage of TCC is the ability to create controlled microstructures within 3D bioprinted scaffolds. When a scaffold is frozen at controlled temperatures, ice crystals with controlled dimensions form, creating a microstructure within the scaffold [18,19,20]. TCC generates a controlled and, if desired, uniform microstructure throughout the 3D-printed object. In contrast, when a large scaffold is frozen from the exterior, the cooling rates during freezing and the direction of ice crystal growth are not uniform throughout the structure and change with distance from the outer surface. In previous works, we demonstrated the ability to control the microarchitecture of scaffolds with freezing [14,21] and used TCC to make 3D printed food with desired micron scale texture for patients with dysphagia [22]. We also demonstrated that the freezing process does not negatively impact the mechanical properties of alginate scaffolds [21].

In summary, the introduction of TCC advances the 3D bioprinting field by offering an effective method of cryopreserving 3D bioprinted scaffolds. As opposed to other methods, which could only cryopreserve small scaffolds, TCC is effective independent of the height of the scaffold.

## 2. Results and Discussion

### 2.1. Printing Multi-Layer Scaffolds

We used the temperature-controlled cryoprinter described previously [16] to print Vero cells encapsulated in an alginate-collagen bioink. Alginate is a popular choice for 3D bioprinting because of its low cost and biocompatibility [23]. In addition, alginate is often used to encapsulate cells for cryopreservation and has demonstrated cryoprotective effects, as it reduces ice crystal formation [24]. Alginate was, therefore, an ideal choice for temperature-controlled cryoprinting, which combines bioprinting and cryopreservation. Since alginate lacks the adhesion sites necessary for cell proliferation, collagen was added to the bioink in a 10:1 ratio.

**Figure 2:**
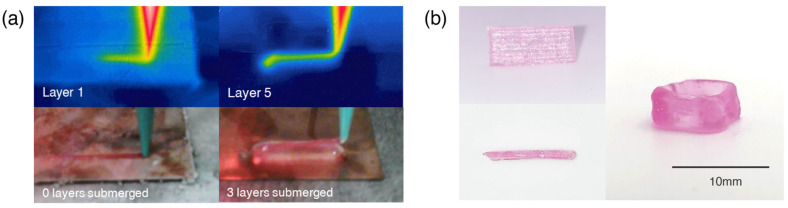
Multi-layer scaffolds printed with temperature-controlled cryoprinting. (**a**) Images taken with a thermal camera during printing demonstrate that the temperature distribution at the nozzle is constant at the first layer and higher layers. (**b**) An eight-layer line and an eight-layer hollow square printed with temperature-controlled cryoprinting.

Temperature-controlled cryoprinting is a promising cryopreservation method for 3D bioprinted scaffolds because each voxel (volume pixel) of bioink is printed under the same thermal conditions. To demonstrate this effect, thermal images of the nozzle were taken during the first layer and fifth layer of a printed scaffold (See Figure 2a). The level of the cooling fluid was such that after three layers were printed the print plate descended and the first layer became submerged in the clear cooling fluid. The three-layer gap prevented the nozzle from touching the cooling fluid and thus freezing and clogging. As demonstrated in Figure 2a, the temperature at the nozzle was maintained even as higher layers were printed. Temperature-controlled cryoprinting was used to print an eight-layer line (Figure 2b) and an eight-layer hollow square (Figure 2b) After printing, the scaffolds were thawed and crosslinked in a CaCl_2_ bath in a process we previously named “freezing-modulated-crosslinking” [16].

### 2.2. Cell Viability during Temperature-Controlled Cryoprinting

The established protocol for cryopreserving mammalian cells is to suspend them in a medium containing 10% DMSO as a cryoprotectant and to slowly cool them at −1 °C/min until they reach temperatures of lower than −60 °C degrees [25,26]. Extensive research during the past fifty years has affirmed the efficacy of slow cooling at −1 °C/min for various mammalian cell types [3]. Although considered the gold standard cryoprotectant, DMSO is toxic to cells at room temperature and so the exposure time should be limited. Ravanbakhsh et al. [9] found that cells encapsulated in a GelMA bioink experienced significant cell death after 30 min of exposure to 10% DMSO. Death from DMSO toxicity can be reduced by exposing it to the cells at 4 °C instead of at room temperature, as DMSO is less toxic at lower temperatures. For long-term cryopreservation, cells cooled to −80 °C should then be cooled to −140 °C using liquid nitrogen. However, studies have demonstrated that for short-term storage, the difference between storing cells at −80 °C and −140 °C is negligible, so for the purposes of this study, the 3D bioprinted scaffolds were stored in a −80 °C freezer for 24 h [27].

#### 2.2.1. Effect of Cooling Rate during 3D Printing

**Figure 3:**
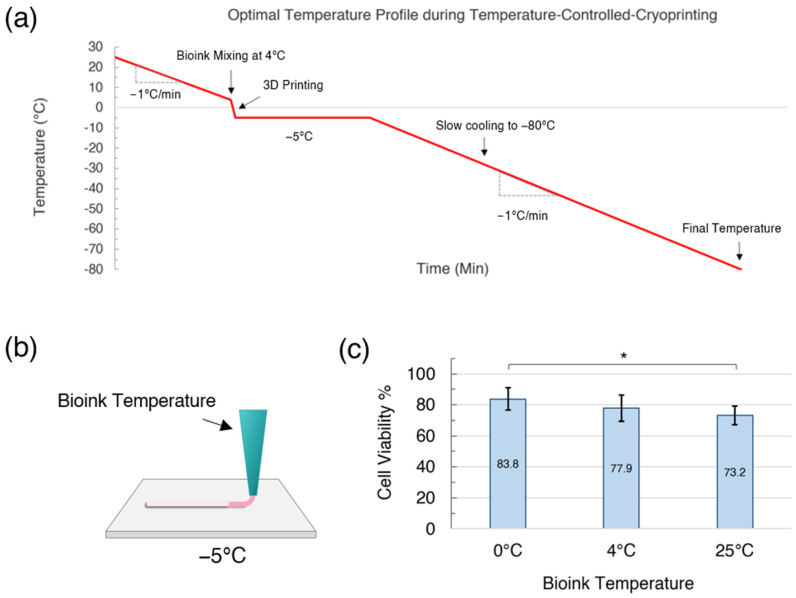
(**a**) An optimal temperature profile for temperature-controlled cryoprinting and subsequent cooling to −80 °C. (**b**) Initial bioink temperature and extrusion onto a −5 °C print plate during temperature-controlled cryoprinting. (**c**) Cell viability rates versus initial bioink temperature. * *p* < 0.05, and error bars represent ± one standard deviation from the mean.

Vero cells were mixed into the alginate-collagen bioink with 10% DMSO using two syringes and a Leuer lock coupler. In this study, we have used the two-step interrupted freezing method, although with the TCC cryoprinting technology the cells could be also cooled at −1 °C/minute from room temperature to −80 °C. However, the printing step of temperature-controlled cryoprinting presented some limitations (See Figure 3a). The freezing point of the bioink was −5 °C; therefore, the print plate needed to remain at −5 °C or lower for cryoprinting to occur. Ravanbakhsh et al. [9] found that using print plate temperatures lower than −5 °C during static cryoprinting reduced cell viability, likely because the cooling rate from the nozzle temperature to the print plate was too rapid. For the purposes of this study, we, therefore, limited our focus to a print plate temperature of −5 °C. A notable feature of this process is that the cells are held at −5 °C until the printing process is completed. Within the cryopreservation literature, this is referred to as a “two-step” freezing protocol. Typically, during two-step freezing the samples are cooled to an initial subzero temperature, held at that temperature for a duration, and then cooled down further to the storage temperature [25,26,28]. Higgins et al. used two-step freezing with a hold temperature of −5 °C to cryopreserve rat embryonic neural cells [29].

To reduce the cooling rate of the bioink closer to −1 °C/min during the printing process, the temperature of the bioink in the nozzle could be lowered, for example, to 4 °C (See Figure 3b). However, lowering the temperature of the bioink increases the viscosity and there becomes an increased risk of cell death from shear stress as the bioink is extruded through the nozzle. Preventing cell death from shear stress is a particular challenge for extrusion-based 3D printing [30]. To test the impact of the bioink temperature in the nozzle, we compared the cell viability for bioink that were cooled to either 25 °C, 4 °C, or 0 °C and then extruded onto a −5 °C print plate (See Figure 3c). Cell death was predicted to be lower when the bioink nozzle temperature was lower because (1) DMSO is less toxic to cells at lower temperatures and (2) the cells were cooled at a slower rate during the 3D printing process. As shown by Figure 3c, cooling the bioink to 0 °C before 3D cryoprinting resulted in the highest cell viability of 83.8 ± 7.19%, while cooling the bioink to 4 °C resulted in a cell viability of 77.9 ± 8.54%, and a bioink temperature of 25 °C resulted in a cell viability of 73.2 ± 6.01%. A one-way ANOVA with a Tukey’s post hoc test found that the drop in cell viability between the bioink cooled to 0 °C and the bioink at 25 °C was statistically significant.

#### 2.2.2. Cell Viability by Layer

**Figure 4:**
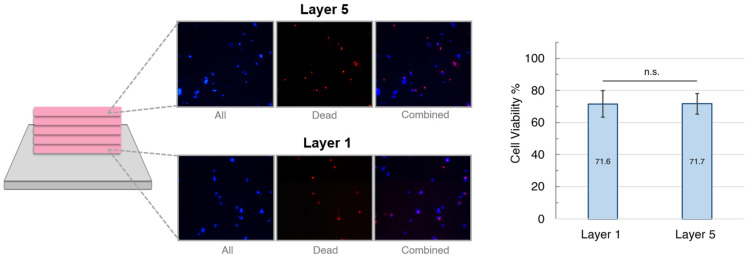
A comparison of cell viability in the first and fifth layers of five-layer scaffolds printed at −5 °C and cryopreserved at −80 °C. Images were taken at the left, middle, and right, along each layer. Fluorescence images include all cells (blue) and dead cells (red). The *p*-value was 0.963 and error bars represent ± one standard deviation from the mean.

A key facet of temperature-controlled cryoprinting is the ability to print multiple layers under the same thermal conditions. Previous literature reported cell viability in scaffolds up to three layers [9]. Using static cryoprinting, printing higher layers either became impossible because the bioink no longer froze or undesirable because cell viability decreased. Because the print plate descends further into the cooling bath as each layer is printed, temperature-controlled cryoprinting presents a method of printing higher layers without compromising cell viability. We compared cell viability between the first and fifth layers for five-layer scaffolds that were cryoprinted and then cooled to −80 °C (see Figure 4). Cells were stained with Hoechst and Propidium Iodide, which stained all cells blue and dead cells red. A one-way ANOVA with a Tukey’s post hoc test was used to assess statistical significance. The average cell viability for Layer 1 was 71.56 ± 8.36% and for Layer 5 was 71.73 ± 6.45%. There was no statistically significant difference in cell viability between the layers with a *p*-value of 0.963. Therefore, we conclude that printing higher layers did not compromise cell viability. Within the field of cryopreservation, cell viability above 70% is generally considered a success [1].

#### 2.2.3. Maximizing Cell Viability during the Stages of 3D Cryoprinting

**Figure 5:**
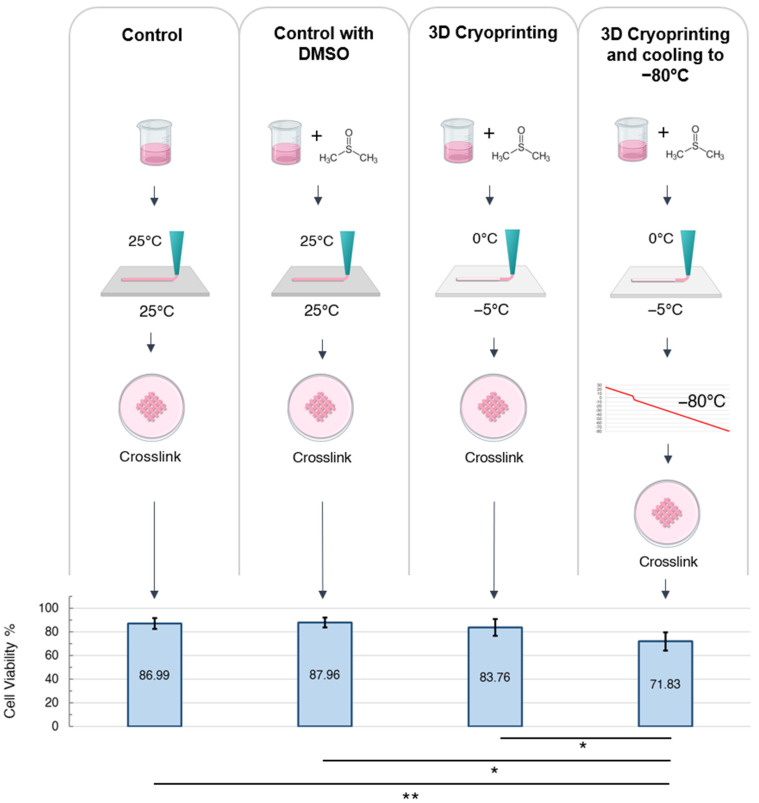
Assessing cell viability during the different stages of the temperature-controlled cryoprinting process. The * *p* < 0.05, ** *p* < 0.01, and error bars represent ± one standard deviation from the mean.

To further elucidate which stages of temperature-controlled cryoprinting caused cell death, we assessed cell viability in one-layer scaffolds after the completion of each step (Figure 5) During the control trial, the cells were mixed into a bioink at room temperature and extruded through the nozzle onto a room temperature plate. They were then crosslinked, washed, and cultured for 24 h. Average cell viability after the control trial was 87.13 ± 4.51%, which is reasonable for 3D bioprinting, suggesting that the process of bioink mixing, extrusion through the nozzle, and crosslinking caused minimal cell death. During the control with DMSO trial, the cells were mixed into a bioink containing 10% DMSO at room temperature. The bioink was then extruded through a nozzle onto a room temperature plate, crosslinked, washed, and cultured for 24 h. The difference in cell viability between the control trial and the control with DMSO trial was not statistically significant, which suggests that exposure to DMSO at room temperature during the bioink mixing and crosslinking stages did not cause significant cell death. This further suggests that the higher cell viability obtained in Figure 3c for bioink cooled to 0 °C before printing was due to the slower cooling rate rather than the reduced exposure to DMSO. During the 3D cryoprinting trial, the cells were cooled to 4 °C and then mixed into a 4 °C bioink containing 10% DMSO. The bioink was then cooled to 0 °C before being 3D printed onto a −5 °C plate. The 3D scaffold was then crosslinked, washed, and cultured for 24 h. The average cell viability for this trial was 83.76 ± 7.19%, which was not a statistically significant difference from the two control trials. During the 3D cryoprinting and cooling to −80 °C trial, the cells were cooled to 4 °C and then mixed into a 4 °C bioink containing 10% DMSO. The bioink was then cooled to 0 °C before being 3D printed onto a −5 °C plate and then the scaffold was cooled at −1 °C/minute to −80 °C. Then, 24 h later, the scaffold was thawed in a 37 °C crosslinker bath, washed, and then cultured for 24 h. The average cell viability was 71.83 ± 7.41%, which was a statistically significant drop from the 3D cryoprinting trial. The results of this experiment suggest that the largest drop in cell viability during TCC occurs as a result of the cells being cooled from −5 °C to −80 °C. This is not unexpected, as the most lethal temperature zone to cells during cryopreservation is between −15 °C and −60 °C [3,4]. The cells traverse this temperature zone twice, once while slow cooling to −80 °C at −1 °C/min and a second time while thawing. Within this temperature zone, ice first forms outside of the cell membrane, increasing the solute concentration and causing osmotic shock. As the temperature continues to lower, intracellular ice forms which penetrates the cell membrane, leading to cell death [3,4]. This two-factor mechanism of cell death can be reduced with an optimized composition of cryoprotectants and post-thawing additives. Therefore, a cell viability greater than 71% during temperature-controlled cryoprinting could likely be achieved with further research. In addition, the bioink composition can also be optimized to achieve cell viability of higher than 87% in the control trials.

## 3. Conclusions

In summary, this study investigated the use of temperature-controlled cryoprinting (TCC) as a method of fabricating and cryopreserving 3D bioprinted scaffolds. The availability of cryopreserved 3D bioprinted scaffolds could allow researchers to create a stockpile of scaffolds and allow labs without 3D bioprinting resources to receive shipments of cell-laden scaffolds from across the world.

We found that TCC could be used to fabricate and cryopreserve 3D bioprinted scaffolds with an average cell viability of 71.64 ± 7.47%. Higher cell viability could likely be achieved by optimizing the bioink composition and the cryoprotectant composition. For example, the use of commercial cryopreservation mediums such as CryoStor^®^ or Unisol™ or the addition of saccharides in the cryopreservation medium have all been shown to significantly increase cell viability after cryopreservation [9,31].

We also found that printing with an initial bioink temperature of 0 °C onto a printing plate at −5 °C resulted in higher cell viability than using a bioink temperature of 4 °C or 25 °C, and our experiments suggested that this was due to the slower cooling rate during printing. Surprisingly, cell exposure to DMSO during TCC did not pose an issue, but further work could also study the impact of reducing DMSO concentration. Future work should also study the long-term cryopreservation of cryoprinted scaffolds on a timescale of weeks or months, although cell viability rates will likely be unchanged [32,33].

In this study, TCC was used to print higher layers than has been achieved with static cryoprinting, and cell death did not increase as higher layers were printed. TCC thus solves an important limitation of static cryoprinting, which is that the cooling rate decreases as further layers are printed and become further away from the print plate. Further studies could investigate the use of TCC for fabricating large-volume scaffolds and even higher layers. While alginate–collagen bioink were used in this study, TCC could likely be used with a variety of bioink, including GelMA, hyaluronic acid, chitosan, or gelatin. TCC could also be used with many different cell types, including human primary cells. In conclusion, TCC is a promising fabrication and cryopreservation technique for 3D bioprinted scaffolds that could positively impact the field of tissue engineering.

## 4. Materials and Methods

### 4.1. Cell Culture

Vero cells were acquired from the University of California Berkeley Cell Culture Facility and were cultured in 5% CO_2_ at 37 °C. The cells were grown in DMEM (Life Technologies, Carlsbad, CA, USA) supplemented with 10% heat-inactivated fetal bovine serum (FBS, Life Technologies, Carlsbad, CA, USA) and 1% Pen Strep (Life Technologies, Carlsbad, CA, USA). Cell passage number was maintained at less than ten.

### 4.2. Bioink Preparation

An amount of 2% (*w*/*v*) alginate was made by dissolving sodium alginate (Spectrum Chemical MFG Corp., New Brunswick, NJ, USA) in DMEM. Next, 1 mL of 2% sodium was mixed with 100 µL of PureCol^®^ EZ Gel Collagen (Advanced Biomatrix, Carlsbad, CA, USA), 100 µL of DMSO (Sigma Aldrich, St. Louis, MO, USA), and 300 µL of cells suspended in Fetal Bovine Serum at 0.5 × 10^6^ cells/mL to create a 1% alginate bioink with 10% DMSO and 0.5 × 10^6^ cells/mL. A lower cell concentration than is typical for 3D bioprinting was used, as cell-to-cell contact can promote intracellular ice formation and thus increase cell death during cryopreservation [34]. The bioink was mixed using two syringes and a Leuer lock coupler at temperatures of either 25 °C or 4 °C. The bioink was then used directly after or cooled to 0 °C.

### 4.3. Temperature-Controlled Cryoprinting

The 3D Scaffolds were printed using a custom-modified temperature-controlled cryoprinter, which has been described in detail in previous works [16]. The cooling bath of the temperature-controlled cryoprinter contained a 45% ethylene–glycol and water solution that was circulated with a Neslab RTE-140 Refrigerated Circulator (Thermo Scientific, Waltham, MA, USA). During 3D printing, the bioink was extruded through an 18-gauge conical nozzle (CML Supply, Lexington, KY, USA) onto the cooled printing plate which then descended further into the cooling bath by the height of the printed layer. The print plate temperature was kept at −5 °C and a printing speed of 2 mm/s was used to print 10 mm lines (See Figure 2). Some 3D scaffolds were then cooled at −1 °C/min to −80 °C in a custom, alcohol-based cooling container in a −80 °C refrigerator. Others were thawed and crosslinked immediately after printing onto the −5 °C plate. The cryopreservation protocol that we have used is known as the interrupted freezing protocol [25,26,28,35,36]. In such a protocol, cells are frozen to a high subfreezing temperature (−5 °C in this study) and kept at that temperature to allow the water in the cell to leave the cell and osmotically equilibrate with the extracellular solution. This removes the possibility for intracellular freezing during the second step of cryopreservation in which the cells are frozen with uncontrolled cooling rates to cryogenic temperatures.

### 4.4. Crosslinking, Thawing, and Cryoprotectant Removal

A 0.5% (*w*/*v*) CaCl_2_ crosslinker was made by dissolving CaCl_2_ dihydrate powder (Fisher Scientific, Waltham, MA, USA) in DMEM. The solution was mixed to homogeneity using a magnetic stir plate. The crosslinker was added at different stages based on the experiment, either directly after 3D printing or after cryopreservation at −80 °C (See Section 2.2.3) Crosslinker at 37 °C was poured over the printed objects and left for 1 min. Then, the crosslinker was removed and the scaffolds were washed three times with DPBS (Sigma Aldrich, St. Louis, MO, USA) to remove excess Ca^+^ ions and dilute the DMSO in the bioink. Scaffolds containing DMSO were then submerged in cell medium and kept at 4 °C for 9 min to allow the DMSO to further diffuse out of the scaffolds. The cell medium was then changed, and the scaffolds were placed in the incubator. The cell medium was changed again at the one-hour and two-hour mark to remove residual DMSO.

### 4.5. Cell Viability Assay

Cells were cultured for 24 h after each experiment as cell death pathways during cryopreservation take 6–24 h to complete [37]. Cell viability assays performed before this 24-h period can thus lead to false positive results. Hoechst/Propidium iodide staining was used to assess cell viability according to the manufacturer’s protocol (Sigma Aldrich, St. Louis, MO, USA). The cell medium was replaced with DPBS, as well as 0.01:1 mL Hoechst and 0.1:1 mL Propidium iodide. The scaffolds were incubated at 37 °C, 5% CO_2_ for 50 min. Thin slices were taken from various parts of the scaffold with a surgical blade, and the slices were then imaged with a Nikon Eclipse TE300 inverted microscope (Nikon, Tokyo, Japan). When imaging the multi-layer scaffolds to compare cell viability between layers, slices were taken at three different locations along the layer, including the left, middle, and right.

### 4.6. Statistical Analysis

All experiments were performed at least in triplicate, and data were presented with ± the standard deviation. A one-way ANOVA was used to compare the means with a Tukey’s post hoc test. The *p*-values of 0.05 and 0.01 were used as the thresholds.

## Data Availability

Data will be made available on request.
